# Extensive calcification of the ligamentum flavum causing cervical myelopathy in a Caucasian woman

**DOI:** 10.1186/s40064-016-3633-z

**Published:** 2016-11-07

**Authors:** Milaine Roet, Jochem K. H. Spoor, M. de Waal, Max J. Kros, Sanjay B. Harhangi, Ruben Dammers

**Affiliations:** 1 Department of Neurosurgery, Erasmus MC, Rotterdam, The Netherlands; 2 Department of Neurology, Albert Schweitzer Ziekenhuis, Dordrecht, The Netherlands; 3 Department of Pathology, Erasmus MC, Rotterdam, The Netherlands; 4 Department of Neurosurgery, Erasmus MC Sophia Children’s Hospital, ‘s Gravendijkwal 230, Office Hs-114, PO Box 2040, 3000 CA Rotterdam, The Netherlands

**Keywords:** Calcification of the ligamentum flavum (CLF), Cervical spine, Caucasian, Myelopathy

## Abstract

Calcification of the ligamentum flavum (CLF) can cause myelopathy due to spinal cord compression. Only several cases in Caucasian patients have been described. Neurological deterioration can only be stopped by surgical decompression. We report a 63-year-old Caucasian woman presenting with progressive pins-and-needles sensations in both hands, worsened by painful paresthesia in both lower extremities. MRI showed a dorsal compressive mass extending from C2 to Th3 vertebrae with myelopathy at the level of C6. A laminectomy was performed, which improved clinical symptoms. Histological examination showed CLF. Early recognition of CLF and early spinal cord decompression are needed to improve neurological outcome.

## Background

Calcification of the ligamentum flavum (CLF) is a rare disease mainly occurring in the cervical spine (Miyasaka et al. 1983). CLF results in posterior spinal cord compression, which may cause myelopathy and successive neurological deficits. It mostly occurs in the Asian population (Ahn et al. 2014). However, several cases in Caucasian patients have been reported (Khan et al. 2005; Ugarriza et al. 2001). The exact pathophysiology is unknown, although findings from multiple case-reports describing CLF suggest that CLF could be a rare manifestation of the deposition of calcium pyrophosphate dehydrate crystals (CPPD) (Berghausen et al. 1987; Brown et al. 1991; Giulioni et al. 2007; Seki et al. 2013). Clinical manifestation usually starts with a loss of vibration, proprioception and functional gait worsened by motor weakness and paresthesia. To distinguish CLF from other causes of dorsal spinal cord compression, radiological imaging, cerebral spinal fluid (CSF) analysis and histological tissue examination are needed. In this report, we describe a case of cervical and thoracic myelopathy due to extensive CLF in a 63-year-old Caucasian woman and its treatment.

## Case report

### Case

**Fig. 1:**
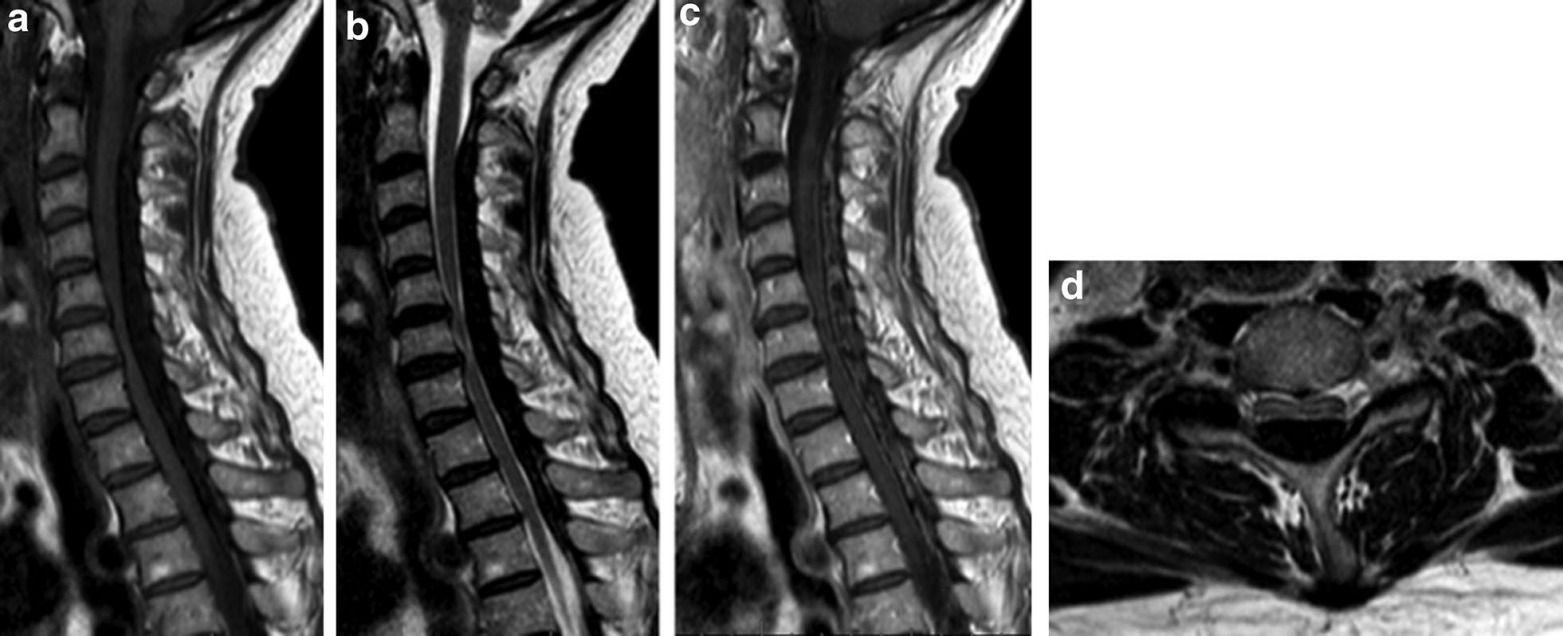
Sagittal MRI in T1- (**a**) and T2- (**b**) weighted sequences. **c** T1-weighted sequence with heterogeneous gadolinium uptake. **d** Extensive spinal cord compression at C6

**Fig. 2:**
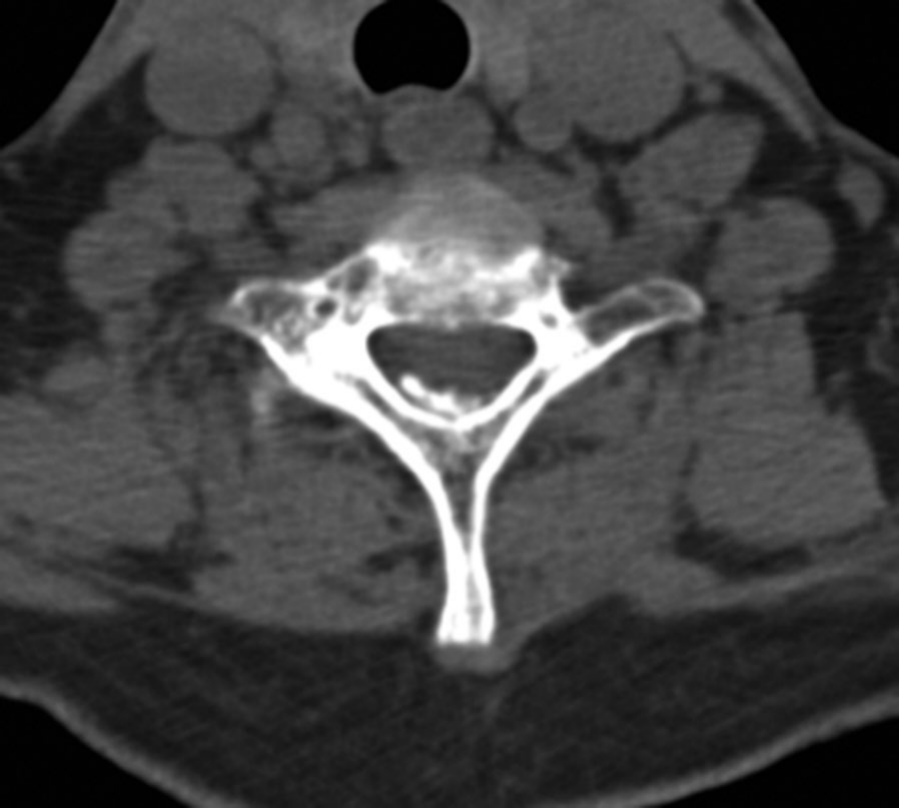
Axial CT-scan showing a hyperdense dorsal intraspinal extradural lesion at C7

**Fig. 3:**
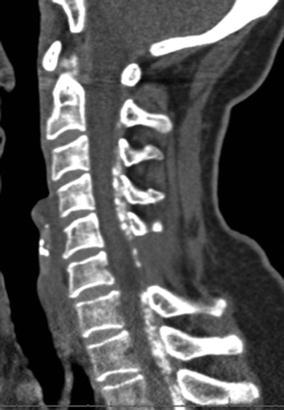
Sagittal CT-scan showing oval nodular densities partially connected with the laminae

A 63-year-old female presented with progressive pins-and-needles sensations in both hands over the past 3 years, worsened by tingling and burning pain in both lower extremities since 4 weeks. Physical examination revealed bilateral hypesthesia of all digits of the hands and loss of vibration sense up to the clavicle following the dermatomes C5–C8. The biceps, triceps and brachioradialis reflexes were all slightly increased. For lower extremities physical examination showed changing hypesthesia on the lateral sides of both legs and feet with a loss in vibration up to the knee. The knee and ankle jerk reflexes were slightly increased with an indifferent plantar reflex bilaterally. There were no changes in muscle tone and Lhermitte’s sign was positive. MRI showed an intraspinal extradural compressive mass extending from C2 to Th3 posterior of the spinal cord. Spinal cord was compressed at the level of C5/6 and C6/7 (Fig. 1). The lesion was hypointense on T1- and T2-weighted MRI sequences and gadolinium contrast showed a heterogeneous uptake. To obtain spinal cord decompression and pathological tissue, a laminectomy at the level of C6 was performed. The lesion consisted of a white, hard granulated substance directly located anterior to the posterior neural arch intertwined with the ligamentum flavum lying on top of a rigid thickened dura mater. Tissue was sent in for histologic examination. CSF was extracted for further analysis. Directly post-laminectomy, neurological examination was as preoperatively. Postoperatively, an additional computed tomography scan (CT-scan) was made to distinguish CLF from ossification of the ligamentum flavum (OLF) and revealed hyperdens oval nodular lesions posterior of the myelum, only partially connected with the laminae (Figs. 2, 3).

### Histology

**Fig. 4:**
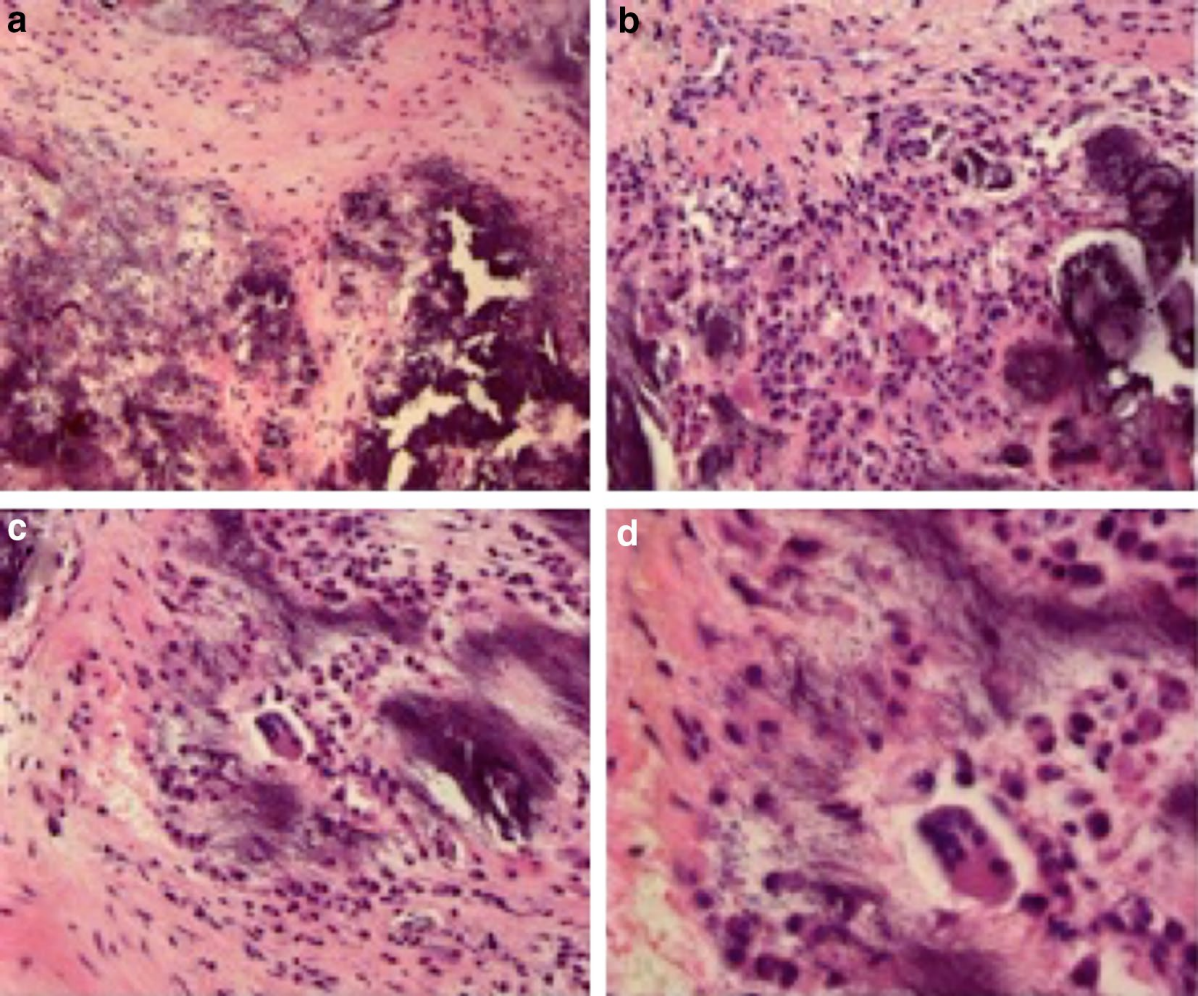
Evacuated lesion with HE staining and magnification ×200 (**a**, **b**, **c**) and ×400 (**d**)

Histology showed ligamentous and cartilage tissue containing extensive dystrophic calcifications intertwined with ligamentum flavum without traces of old blood or mature bone formation (Fig. 4). Histologically there were no signs of lymphoma, nor traces of mycobacterium tuberculosis, other bacteria or fungi. CSF analysis showed no indications of lymphoma or infection either.

### Follow-up

**Fig. 5:**
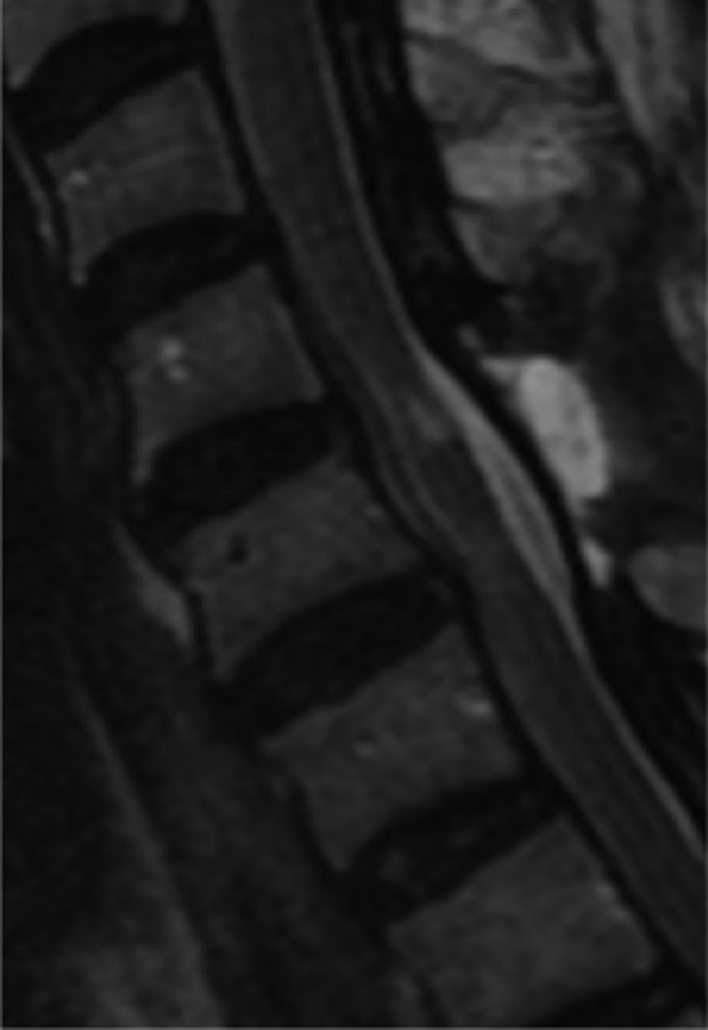
Sagittal MRI in T2-weighted sequence showing decompression of the spinal cord after laminectomy

One-month MRI follow-up showed no progression of the lesion and a clear decompression of the spinal cord at the operated level (Fig. 5). Eight months later, hypesthesia of all digits of the right hand and in lesser extend of the left hand had become less, reflexes of her upper extremities normalized. The tingling sensations and reflexes in her lower extremities persisted.

## Discussion

Dorsal spinal cord compression can be caused by different pathological conditions. MRI imaging is important to differentiate from neoplasm, meningioma, lymphoma and chronic calcified epidural hematoma (Boutarbouch et al. 2008; Matsui et al. 2014; Seo et al. 2014; Zhong et al. 2011). To exclude pachymeningitis, CFS analysis is needed (Senapati et al. 2014). Sagittal CT-imaging revealed oval nodular densities, partially connected with the laminae. This distinguishes CLF from OLF since OLF arises from the lamina and moves inward towards the midline (Miyasaka et al. 1983). The exact pathophysiology of CLF is yet to be discovered. Seki et al. described a large calcified mass in the ligamentum flavum level L4/5 and after resection, additional analyses showed the mass mostly consisted of calcium triphosphate and calcium phosphate with degenerative changes possibly involved in the formation of this mass (Seki et al. 2013). Other reports describing cervical CLF also mention nodular deposition of calcium pyrophosphate dehydrate crystals in the ligamentum flavum (Berghausen et al. 1987; Brown et al. 1991; Cabre et al. 2001). CPPD crystal formation can be sporadic or hereditary and may be related to metabolic diseases such as hypophosphatemia, hypothyroidism, hyperparathyroidism and hemochromatosis (Berghausen et al. 1987; Riccio et al. 2016). Calcium crystal deposition in the ligamentum flavum seems to progress with a reduction in elastic fibers, an increase in collagen fibrils and migration of hypertrophic chondrocytes (Mwaka et al. 2009). This indicates that CPPD may be a consequence of degenerative changes in the ligamentum flavum, which might explain the broad extension of the lesion in our case. Recent research shows that mechanical load may be a contributing factor to CLF. They found that mechanical load induces the differentiation of ligamentum flavum cells leading to calcium depositions (Chao et al. 2016).

The only treatment for CLF is surgical decompression of the spinal cord (Ahn et al. 2014; Delgado-Lopez et al. 2007; Inoue et al. 2013; Khan et al. 2005; Sun et al. 2014; Yuan et al. 2014). The right method of decompression, we believe, is laminectomy. A retrospective study of thoracic OLF showed that laminectomy with fusion had a better outcome than laminoplasty (Li et al. 2006). Such a large study has not been executed for CLF. One study shows good clinical outcome after endoscopic partial laminectomy (Yabuki and Kikuchi 2008). Since the diagnoses was uncertain and we considered it to be a lymphoma we decided to perform a laminectomy of only C6 first in order to decompress the spinal cord where it showed myelopathy and to obtain tissue for histology. Since clinical outcome improved, we, in consolation with our patient, decided to only further decompress the spinal cord should neurological deterioration occur in the future. Clinical symptoms were mild and stable and no further decompression was there for deemed necessary.

## Conclusion

Although rare, CLF should be considered as a possible diagnosis of extensive dorsal spinal cord compression. It is important to recognize and treat CLF at an early stage, since timely decompression results in better neurologic outcome.

## Abbreviations

CLF: calcification of the ligamentum flavum
CSF: cerebrospinal fluid
CT-scan: computed tomography scan
MRI: magnetic resonance imaging
OLF: ossification of the ligamentum flavum
CPPD: calcium pyrophosphate dihydrate crystal deposition disease
